# Research on the development of Chaozhou woodcarving from the perspectives of grounded theory and biomechanics

**DOI:** 10.1371/journal.pone.0341450

**Published:** 2026-02-18

**Authors:** Liefeng Li, Fan Wu, Wangshuang Zang, Lin Fan

**Affiliations:** 1 Guangzhou University of Software, Guangzhou, China; 2 School of Media and Art Design, Wenzhou Business College, Wenzhou, China; Macau University of Science and Technology, MACAO

## Abstract

This study establishes a novel Bio-Cultural Heritage Design Framework that integrates grounded theory and biomechanics to address the sustainable development of traditional crafts in the digital era. Using Chaozhou woodcarving as a case study, we constructed a hierarchical demand system through a three-stage coding analysis of 1,250 online reviews, 381 survey responses, and expert interviews, identifying biomechanical utility (A1, 40% weight) as the dominant factor. Key sub-criteria—ergonomic compatibility (C12, 0.21 weight), material durability (e.g., nanmu wood with elastic modulus ≥8 GPa), and structural stability (C22, 0.15 weight)—were translated into AI design constraints via Analytic Hierarchy Process (AHP). AI-generated prototypes (e.g., Sample 15, score 4.53/5) demonstrated superior biomechanical performance, validated by finite element analysis (stress distribution <10 MPa) and user evaluations, outperforming traditional designs by 40–47%. This framework bridges cultural authenticity with scientific rigor, offering actionable strategies to optimize functional reliability while preserving heritage aesthetics, thereby addressing the critical challenge of balancing craft preservation with modern market demands.

## 1. Introduction

In the digital era, traditional craftsmanship, a cornerstone of Intangible Cultural Heritage (ICH), faces an existential crisis. As modern manufacturing technologies and shifting consumer lifestyles reshape the global market, many heritage crafts struggle to maintain their relevance and functionality in daily life [1–3]. The tension between preserving cultural authenticity and adapting to modern utility has created a bottleneck for sustainable development. While government policies often focus on preservation through archival documentation (e.g., National Intangible Cultural Heritage lists) and financial subsidies for representative inheritors, there is an urgent need for innovative strategies that can reintegrate these crafts into the contemporary consumer ecosystem without diluting their artistic essence.

This challenge is particularly evident in Chaozhou Woodcarving, a premier school of folk art from Guangdong, China. Renowned for its distinct multi-layered “hollow carving” (Tongdiao) technique and rich gold lacquer finish, Chaozhou Woodcarving dates back to the Tang Dynasty and reached its zenith during the Qing Dynasty [4]. Historically, it served as intricate architectural decoration for temples and ancestral halls, as well as functional religious artifacts. However, in the modern context, these elaborate forms often clash with the minimalist aesthetics and ergonomic requirements of contemporary living spaces. This incompatibility has led to a shrinking market share and reduced economic viability, which discourages young talent from entering the profession. Consequently, to mitigate market risks, producers often retreat to repetitive reproduction of classic patterns, resulting in severe product homogenization and a further disconnect from mass-market utility [5,6].

The existing body of literature has extensively documented the artistic characteristics and cultural symbolism of Chaozhou Woodcarving [7–9], and recent studies have identified the market challenges it faces [10–13]. Furthermore, emerging research has begun to explore the application of digital technologies, such as AI-Generated Content (AIGC), in art and design [14,15]. However, a critical gap remains: existing studies predominantly focus on aesthetic digitization or visual style transfer, often neglecting user-centered design considerations such as ergonomic constraints and functional reliability. Currently, few frameworks effectively translate empirical consumer insights—specifically latent needs for comfort and durability—into actionable, scientifically grounded design constraints for AI.

To bridge this gap, this study establishes and validates a novel Bio-Cultural Heritage Design Framework. Unlike traditional approaches, this framework integrates Grounded Theory, Biomechanics, and Artificial Intelligence. Its core logic is to transform qualitative user feedback into quantitative biomechanical parameters (e.g., ergonomic curvature, stress thresholds), which then serve as objective constraints for AIGC tools. By doing so, we aim to create a replicable pathway that not only preserves the visual identity of Chaozhou Woodcarving but also optimizes its functional reliability for the modern market.

## 2. Materials and methods

The following two methods were mainly used in this study.

### 2.1. Research methods and process description

Grounded theory, developed by Glaser and Strauss in 1967 [16], is a qualitative research method that constructs theory from empirical data. It involves generating concepts from data, comparing data and concepts, developing theoretical concepts, establishing connections between concepts, and systematically coding data hierarchically [17]. The research process does not start with preconceived theoretical assumptions but emphasizes the generative construction of theory through continuous concept development [18,19]. In this study, grounded theory was used to analyze the characteristics of Chaozhou Woodcarving Art and construct a system of sustainable development demand elements. The process included data collection from online reviews, interviews, and questionnaires, followed by open, axial, and selective coding to identify key concepts and themes. Theoretical sampling was conducted until theoretical saturation was achieved, ensuring the theory was derived directly from the data.

AIGC (Artificial Intelligence Generated Content) refers to the generation of text, images, or audio/video content based on prompt information. It leverages technologies such as Natural Language Processing (NLP), Generative Adversarial Networks (GANs), and diffusion models to create new content [20,21]. AIGC has brought significant transformation to content creation and production, offering new opportunities and challenges to the creative industry. In the design field, AIGC tools such as large language models like ChatGPT and image generation tools like Midjourney and Stable Diffusion are widely used. They are applied to improve repetitive tasks and generate large-scale solutions at low cost in a short time, mainly serving as a source of inspiration. For example, Zhu applied Generative Adversarial Networks (GANs) to achieve high-quality replication and restoration of calligraphy art. This method not only preserved the original style of the artworks but also improved efficiency and reduced costs, offering a novel solution for cultural heritage preservation [22].

**Fig 1:**
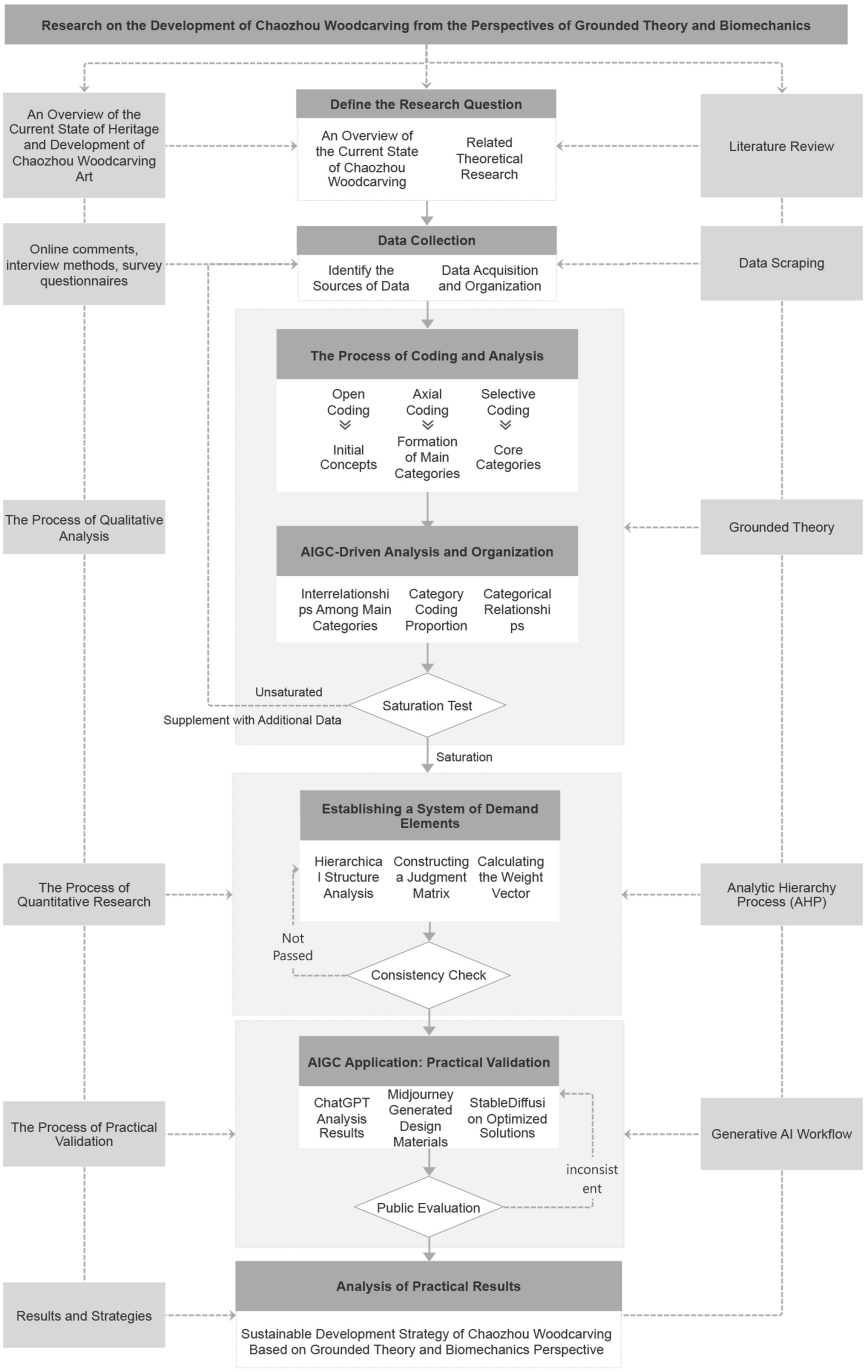
Research roadmap.

In this study, AIGC was used to generate design solutions for Chaozhou Woodcarving. Specifically, AI tools such as Midjourney and Stable Diffusion were employed to create prototypes based on the weighted criteria derived from the Analytic Hierarchy Process (AHP) [23]. These AI-generated designs were then evaluated through public voting, demonstrating their potential to meet consumer demands while preserving the cultural heritage of Chaozhou Woodcarving. The research process can be seen in Fig 1 Research Roadmap.

### 2.2. Data collection and organization

#### 2.2.1. Participant demographics.

The survey yielded 381 valid responses with a balanced gender distribution (Males: 182, 47.8%; Females: 199, 52.2%). The age structure was predominantly young to middle-aged, with 86.4% of participants aged between 18 and 44 (18–24: 128; 25–34: 120; 35–44: 81), while those under 18 (5) and over 45 (37) constituted smaller portions. In terms of occupation, white-collar and office workers formed the majority (253, 66.4%), followed by homemakers (55), freelancers (38), students (17), and others (18). Monthly income distribution showed that middle-to-high income groups (≥5,000 CNY) accounted for 55.4% of the sample (5,000–10,000 CNY: 122; > 10,000 CNY: 89), indicating a strong potential purchasing power for cultural products. Informed consent was obtained from all participants prior to the survey and interviews (S2 File).

#### 2.2.2. Data collection channels and procedures.

To address the current development challenges of Chaozhou Woodcarving, this study collected and organized data from the objective market perspective, obtaining research data from three main channels. One of the channels was online reviews from third-party platform users. Using Python, a web crawler program was developed to collect reviews related to Chaozhou Woodcarving products from major e-commerce platforms such as Taobao, JD.com, and Pinduoduo. The content obtained included product types, images, and review texts. As of November 2024, a total of 2,247 reviews related to “Chaozhou Woodcarving” were collected, with 1,250 valid reviews remaining after removing those deemed to be distracting (S1 File).

Product evaluations were sourced from official platforms, primarily the Guangdong Provincial Museum’s Tmall flagship store and the National Museum of China’s JD.com flagship store. The Guangdong Provincial Museum’s store offered 14 Chaozhou Woodcarving products, including creative bookmarks, paper-cut lamps, stickers, and DIY ornaments, with 37 valid reviews collected. The National Museum of China’s JD.com store, using “wooden” as the search tag, yielded 216 data entries, mainly featuring cultural and creative decorations and tabletop ornaments.

In addition, interviews and questionnaires were conducted with potential users. After organizing the online review data, a survey questionnaire was created based on the product types sold through the main official channels. These were conducted both offline in the Guangdong-Hong Kong-Macao Greater Bay Area and online, targeting young and middle-aged consumers to gather data on the desired forms of Chaozhou Woodcarving products. See Table 1 for details.

**Table 1:** Survey data and information on chaozhou woodcarving products.

User Category	Data Channel	Data Source	Number of Valid Comments/Interviews	Typical Products
Target Users	Third-Party Platforms	Taobao	146/entries	Wooden crab basket
		JD.com	561/entries	Wooden carving sword
	Official Channels	Pinduoduo	543/entries	Hand carved ornaments
		Guangdong Provincial Museum Flagship Store	37/entries	Chaozhou Wooden Carving Shrine
		National Museum of China Flagship Store	216/entries	Creative Woodcarving Ornaments
Potential Users	Online/Offline	Surveys/Interviews	381/responses	–

To ensure the quality and validity of the dataset, a rigorous data cleaning process was implemented following the initial collection. This process involved: (1) removing duplicate entries and automated spam; (2) filtering out reviews that were irrelevant to the product’s attributes, such as comments solely concerning logistics, packaging, or customer service; and (3) excluding non-descriptive, low-information content (e.g., “good,” “received”). This procedure refined the initial dataset of 2,247 reviews to 1,250 valid entries for analysis. Furthermore, sourcing data from multiple platforms (Taobao, JD.com, Pinduoduo) served to mitigate potential sampling bias from any single user population.

Data Compliance Statement: The collection and analysis of online reviews from e-commerce platforms (Taobao, JD.com, and Pinduoduo) were conducted in compliance with the respective platforms’ terms and conditions. The data collected were publicly available user-generated content, and no private or personally identifiable information was accessed or used. The study focused solely on the textual content of the reviews for academic research purposes. For the survey and interview data, informed consent was obtained from all participants prior to their involvement, as stated in the ‘Informed Consent Statement’ within the manuscript.

### 2.3. Coding and analysis process

#### 2.3.1. Grounded theory methodology.

This study adopted the systematic procedures of Grounded Theory, specifically the Strauss and Corbin approach [24], rather than simple thematic analysis. The goal was not merely to categorize themes, but to construct a substantive theory that explains the hierarchical and causal relationships between user demands and design elements. The process involved three rigorous stages:

(1) Open Coding: Deconstructing raw data into initial concepts (e.g., labeling user feedback on “grip comfort” as specific ergonomic indicators).
(2) Axial Coding: Identifying relationships between categories to form main categories (e.g., establishing how Biomechanical Utility functionally supports Consumption Value).
(3) Selective Coding: Integrating all categories around the core category to build the final demand system.

This theoretical construction process ensures that the resulting framework is not just a list of requirements, but a structured system of interacting variables, validated manually by researchers to ensure integrity.

#### 2.3.2 Implementation and coding results.

Given the volume of raw data, the coding process was primarily managed using NVivo and Excel software. To enhance rigor and mitigate potential researcher bias, ChatGPT was employed as a supplementary tool for cross-validation. Initially, the research team performed manual open coding to generate 42 initial concepts (S3 File). Subsequently, the raw data and these initial concepts were presented to ChatGPT to generate thematic classifications. The AI-generated themes were then systematically compared against the manually derived concepts by the researchers. This comparative process helped to refine category definitions and identify nuanced relationships, leading to the final set of 33 initial concepts. Crucially, all final coding decisions, the categorization into nine basic categories, and the construction of the theoretical framework were performed and validated manually by the researchers to ensure integrity (S4 File).

This leads to the clear identification of 33 initial concepts, such as a1 Ergonomic Woodcarving Ornament, a2 Ergonomic Woodcarving Handle Piece, a3 Ergonomic Woodcarving Jewelry,..., a31 Stress Relief Effect, a32 Emotional At-traction, and a33 Willingness to collect. Immediately following this, nine basic categories are derived: C1 Form and Quality Fit, C2 Material Mechanics and Craftsmanship, C3 Sensory Feedback during Use,..., C7 Value Judgment, C8 Educational Function, and C9 Psychological Needs. To avoid subjective interference, the original sentences are listed alongside their corresponding concepts and categories, as detailed in Table 2.

**Table 2:** Open encoding.

Initial Category	Initial Concept	Original Statement (Example)
C1 Form and Ergonomics Fit	a1 Ergonomic Woodcarving Ornament a2 Ergonomic Woodcarving Handle Piece a3 Ergonomic Woodcarving Jewelry a4 Ergonomic Woodcarving Bead String a5 Ergonomic Woodcarving Creative Item a6 Ergonomic Compatibility	The unique texture and lightweight design of the solid wood make it an ideal ergonomic ornament, fitting comfortably in various settings. The vivid carving and compact size create an adorable ergonomic handle piece, perfect for enhancing the comfort of a tea table setup. The beautiful bead string features ergonomic jewelry design with a size that fits comfortably when worn, ensuring both aesthetics and comfort. The lightweight design of the bird figurine ensures it has good ergonomic compatibility, making it easy to place and reposition. The exquisite bird figurine, with its palm-sized dimensions, serves as an ergonomic decorative item that fits well in various spaces.
C2 Material Mechanics and Craftsmanship	a7 Carving Craftsmanship and Material Strength a8 Wood Material Mechanical Properties a9 DIY Creation and Material Process ability a10 Craftsmanship Difficulty and Material Mastery	The beads exhibit uniform size and smoothness, showcasing excellent carving craftsmanship that enhances the material’s strength and provides a superior tactile experience. Carving the hairpin required three hours, demonstrating the material’s process ability and the fine craftsmanship achieved, which I appreciate and will repurchase for its quality. Mastering the material’s properties through skillful manual carving is quite challenging, and completing it signifies a meaningful achievement in craftsmanship difficulty and material mastery.
C3 Sensory Feedback during Use	a11 Tactile Experience during Use a12 Aroma Experience during Use a13 Visual Appeal during Use a14 Atmosphere Created by the Product	The aged wood has a faint scent of sandalwood and high oil content, giving a strong sense of weight when held. Each bead is plump, round, and smooth, providing a great tactile experience during use. I’m extremely satisfied with the material’s tactile experience and the color tone. The small-leaf rosewood has a wonderful aroma, and when I add a few drops of my essential oil, the sensory experience during use is just perfect.
C4 Aesthetic Style	a15 Design Aesthetics a16 Artistic Style a17 Creativity and Novelty	The seller was really great, and I picked a nice string of beads as a gift for someone. It’s a Chinese zodiac bracelet, very traditional and Chinese in style. I’m very satisfied! The beads are very beautiful, with unique shapes. The item has a particularly trendy and fashionable style, and it looks great.
C5 Cultural Symbolism	a18 Cultural Symbolic Elements a19 Feng Shui Implications a20 Story Connotations	Displaying a peach wood sword at home not only demonstrates the owner’s respect for traditional culture but also serves as a feng shui ornament, adding a touch of classical charm to the living environment. It’s really quite nice. I’ve received the Wen Chang Pagoda. I bought it to help my child with their studies, hoping they will achieve good grades and get into a good university.
C6 Shopping Attributes	a21 Gift-Giving Purpose a22 Product Class a23 Elegant Packaging a24 After-Sales Service	After being sealed for a few days and then taken out for handling, I simply love it. The beads are oily and smooth with plenty of gold stars, and the gifts included are also rich and practical. The birthday gift for my friend has arrived, and I’m very satisfied. The craftsmanship is very meticulous and not at all rough.
C7 Value Assessment	a25 Value Assessment a26 Price Reasonableness a27 Cost-Effectiveness	The bead string is really good, and I’m very satisfied at this price point. It has a high aesthetic value and looks really great when worn. The cost-effectiveness is truly exceptional.
C8 Educational Function	a28 Educational Inspiration a29 Heritage Carrier a30 Hands-on Practice	It was so much fun! I sat down with my kid for the whole afternoon with the tools.
C9 Psychological Needs	a31 Stress-Relief Effect a32 Emotional Appeal a33 Collecting Intention	Whittling wood is very relaxing. Friends who are interested should definitely give it a try. I spotted this adorable little figurine at first glance and bought it as a decoration for my girlfriend. She really loves it! It’s such a cute little cat! I believe it will bring good luck for the New Year!

Based on open coding, the main categories were summarized and the relationships between them were clarified [25]. A1 Biomechanical Utility Value, A2 Cultural Aesthetic Value, A3 Consumption and Social Value, A4 Educational and Heritage Value, and A5 Emotional Fulfillment Value. Specifically, A1 involves form and quality compatibility, material and craftsmanship, and Sensory Feedback during Use, which together determine a product’s practical usability and user experience. A2 is reflected in cultural symbolism and aesthetic style, showcasing the cultural heritage of Chaozhou woodcarving through creative designs. A3 focuses on shopping attributes and value assessment, such as gift-giving purposes and packaging quality, which influence purchasing decisions. A4 is primarily about educational functions and heritage transmission, with woodcarvings serving as carriers for cultural education and skill inheritance. A5 includes stress relief, emotional appeal, and collecting intentions, with cute or unique designs attracting consumers and evoking a sense of affection. These categories are interwoven, forming consumers’ comprehensive understanding of Chaozhou woodcarving and providing a basis for heritage strategies. For specific classification, see Table 3.

**Table 3:** Axial coding of main categories and corresponding initial categories.

Main Category	Initial Category	Original Statement (Example)
A1 Biomechanical Utility Value	C1 Form and Ergonomics Fit C2 Material Mechanics and Craftsmanship C3 Sensory Feedback during Use	The design is unique and the texture is great, making it particularly suitable for placement in the car. The color matches the interior, and the size is just right. The craftsmanship is exquisite, and the gifts included are also great. I bought it for my daughter, and it was a very satisfying purchase. The product has a good sensory feedback during use, making it a delightful experience overall.
A2 Cultural Aesthetic Value	C4 Aesthetic Style C5 Cultural Symbolism s	The Chinese-style zodiac bracelet is very beautiful when worn, and the size fits perfectly. The craftsmanship is exquisite, and I hope it will bring my child good luck in their studies. The peach wood sword is well-made. I got it to protect my home. The fine craftsmanship gives me peace of mind.
A3 Consumption and Social Value	C6 Shopping Attributes C7 Value Assessment	The craftsmanship is exquisite, and the gifts included are also great. I bought it for my daughter, and it was a very satisfying purchase. I bought it as a gift for my dad. He said it’s very worth it at this price and he particularly likes it.
A4 Educational and Heritage Value	C8 Educational Function	It’s really great, worth the money, and relaxing. Playing with my child was amazing. We not only learned how to use our hands but also enjoyed many vivid stories together.
A5 Emotional Fulfillment Value	C9 Psychological Need	It’s very nice and soothing. The simple expression of the little cat captures a sense of contentment, reminding us that happiness can be found in simplicity.

Selective coding involves establishing connections between main categories and other categories, and deeply analyzing their relationships. This process constructs a systematic and complete framework reflecting the original data categories [26]. Through grounded theory, the study identified the interrelatedness of the five main categories: A1 Biomechanical Utility Value, A2 Cultural Aesthetic Value, A3 Consumption and Social Value, A4 Educational and Heritage Value, and A5 Emotional Fulfillment Value. These categories influence and interact with each other.

A1 serves as the foundation, enhancing A2 through high-quality materials and exquisite craftsmanship, which better present cultural symbolism and aesthetic styles. It also supports A3, as products with excellent utilitarian experience have advantages in shopping attributes and value assessment, making them suitable for social purposes. A1 promotes A4, allowing consumers to experience the charm of woodcarving in practice, facilitating its inheritance. A2 adds unique charm to the product, enriching A5 by stimulating consumers’ affection and desire to collect. A3 is influenced by the other categories and affects the product’s market positioning. A4 enhances consumers’ understanding of cultural aesthetics and strengthens their emotional identification with woodcarving. A5 permeates the entire consumption process, driving consumers’ choices and evaluations of products and influencing the realization of the other categories. For details, see Table 4: Selective Coding.

**Table 4:** Selective encoding.

Typical Relationship Structure	Relationship Structure	Original Statement (Example)
Biomechanical Utility Affects Emotional Evaluation	A1 Biomechanical Utility Value (Perceived Functional Quality) → A5 Emotional Fulfillment Value (Perceived Emotional Quality)	The craftsmanship is exquisite and suitable as a decoration; it looks very classy in the living room. I initially thought this piece would be larger, but upon receiving it, I realized the size is not quite right. It feels awkward to hold, and I’m not very fond of it.
Emotional Drive Influences Social Choice	A5 Emotional Fulfillment Value (Perceived Emotional Quality) → A3 Consumption and Social Value (Perceived Social Quality)	This little bird pendant is so cute!! I hope it brings me good luck, and I’ll buy a few more to give to my friends.
Biomechanical Utility Feedbacks Social Interaction	A1 Biomechanical Utility Value (Perceived Functional Quality) → A3 Consumption and Social Value (Perceived Social Quality)	I carved this for my girlfriend for the first time, and she couldn’t stop praising it. We even discussed together how to display it better. Ha haha, I’ll buy another one to play with her.
Cultural Aesthetics Enrich Emotional Connotations	A2 Cultural Aesthetic Value → A5 Emotional Fulfillment Value	The solemn and beautiful deity statue, worshipped with a sincere heart, not only looks beautiful at home but also helps me maintain a compassionate mind and stay away from negative thoughts!
Educational Heritage Enhances Cultural Aesthetics	A4 Educational and Heritage Value → A2 Cultural Aesthetic Value	I bought it to play with my child, and only then did I truly understand the stories behind the woodcarvings. Now, when I look at those woodcarvings again, I find their beauty even more profound.

### 2.4. Establishment of demand element system model and parameter calculation

Following the progressive coding process from open coding to axial coding and then selective coding, a questionnaire survey was conducted among potential users regarding the modern inheritance and development factors of Chaozhou woodcarving, with a total of 381 responses collected to test the theoretical saturation. The results showed that the close relationships among the five main categories, as well as the interconnections among the factors within each category, were also reflected in these data. No new categories emerged, and there was no impact on the core category concepts that had been identified. This is consistent with the principle of theoretical saturation.

Following the hierarchical progression of category coding, we have established the current needs system for Chaozhou woodcarving. The main categories serve as the criterion layer, while the initial categories form the sub-criterion layer. We have removed identical or synonymous initial concepts to avoid redundancy and integrated them into representative semantic phrases, which are matched one-to-one to build the current needs system for Chaozhou woodcarving, as shown in Table 5. The five criterion layers cover all the core value areas involved in the development of Chaozhou woodcarving in today’s society. These layers don’t exist in isolation; they’re closely connected. This system can provide practical guidance parameters for the development of Chaozhou woodcarving.

**Table 5:** Chaozhou woodcarving demand element system.

Criterion Layer	Sub-Criterion Layer	Representative Semantic Phrases (Positive – Negative)
A1 Biomechanical Utility Value	C1 Form and Ergonomics Fit	C11 Appropriate Size – Inappropriate Size C12 Ergonomically Comfortable – Uncomfortable
	C2 Material Mechanics and Craftsmanship p	C21 High-Quality Material – Poor Material C22 Exquisite Carving – Coarse Carving C23 DIY Feasible – Restricted
	C3 Sensory Feedback during Use	C31 Good Tactile Experience – Poor Tactile Experience C32 Pleasant Scent – Irritating Scent C33 Attractive Appearance – Dull Appearance C34 Atmosphere Harmony – Discordant
A2 Cultural Aesthetic Value	C4 Aesthetic Style	C41 Aesthetic Design – Mediocre Design C42 Diverse Style – Monotonous Style C43 Innovative Creativity – Outdated
	C5 Cultural Symbolism	C51 Rich Cultural Connotations – Lack of Cultural Connotations C52 Feng Shui Symbolism Recognized – Not Recognized C53 Deep Story Connotations – Shallow
A3 Consumption and Social Value	C6 Shopping Attributes	C61 Suitable for Gifts – Unsuitable for Gifts C62 Clear Product Quality – Vague Product Quality C63 Elegant Packaging – Poor Packaging C64 Comprehensive After-Sales Service – Inadequate After-Sales Service
	C7 Value Assessment	C71 Accurate Value Assessment – Deviation in Value Assessment C72 Reasonable Price – Unreasonable Price C73 High Cost-Effectiveness – Low Cost-Effectiveness
A4 Educational and Heritage Value	C8 Educational Function	C81 Effective Educational Inspiration – Ineffective Educational Inspiration C82 Prominent Heritage Carrier – Not Prominent Heritage Carrier C83 Simple Operation – Complex Operation C84 Complete Practical Guidance – Missing Practical Guidance
A5 Emotional Fulfillment Value	C9 Psychological Needs	C91 Significant Stress Relief – Weak Stress Relief C92 Pronounced Emotional Relaxation – Insignificant Emotional Relaxation C93 Strong Emotional Resonance – Weak Emotional Resonance C94 Deep Cultural Empathy – Shallow Cultural Empathy C95 Collection Preference Match – Mismatch

To assess the criteria, sub-criteria, and representative semantic phrases within the system, we employed a Likert scale using a comparative rating method. We invited nine experts, including practitioners of Chaozhou woodcarving, as well as masters and doctoral students in design and art, along with relevant researchers, to form a rating panel. Selection criteria for these experts included a minimum of 5 ~ 10 years of professional experience in their respective fields and holding professional titles (S5 File). Using a scale of 1–9 and their reciprocals for judgment [27], we first collected pairwise comparison ratings from the nine experts for the criteria, sub-criteria, and semantic phrases. We then calculated the mean values of these ratings to construct matrices. The product of each row was calculated to find the geometric mean, and the results were normalised to obtain the weights (see Table 6 for an example of the criteria weights). Finally, the weights were integrated and ranked, as shown in Table 7, which details the weights of each level of needs elements.

**Table 6:** Weight calculation table for criterion layer.

	A1	A2	A3	A4	A5	Product	Geometric Mean	Weight
A1	1.00	2.50	3.00	2.80	3.00	63.00	2.29	0.40
A2	0.40	1.00	2.00	1.80	2.00	2.88	1.24	0.21
A3	0.33	0.50	1.00	0.90	1.20	0.18	0.71	0.12
A4	0.36	0.56	1.11	1.00	2.50	0.56	0.89	0.15
A5	0.33	0.50	0.83	0.91	1.00	0.12	0.66	0.11

**Table 7:** Detailed list of weights at various levels.

Criterion Layer	Weight	Sub-Criterion Layer	Weight	Representative Semantic Phrases (Positive – Negative)	Weight
A1	0.40	C1	0.37	C11	0.29
				C12	0.21
		C2	0.12	C21	0.11
				C22	0.15
				C23	0.06
		C3	0.10	C31	0.08
				C32	0.04
				C33	0.03
				C34	0.02
A2	0.21	C4	0.10	C41	0.39
				C42	0.24
				C43	0.15
		C5	0.09	C51	0.10
				C52	0.07
				C53	0.04
A3	0.12	C6	0.07	C61	0.36
				C62	0.23
				C63	0.10
				C64	0.15
		C7	0.06	C71	0.05
				C72	0.07
				C73	0.03
A4	0.15	C8	0.05	C81	0.50
				C82	0.27
				C83	0.15
				C84	0.08
A5	0.11	C9	0.05	C91	0.49
				C92	0.26
				C93	0.08
				C94	0.14
				C95	0.03

To ensure the reliability of the expert judgments, the consistency ratio (CR) was calculated for each judgment matrix. For the primary criterion layer matrix (Table 6), the calculated CR was 0.0591. As this value is below the standard threshold of 0.10, it confirms that the pairwise comparisons were logically consistent and the resulting weights are valid for subsequent analysis.

## 3. Integrating AI into design practice and strategy analysis

While grounded theory is effective in identifying what consumers demand, it does not quantify the relative importance of these, often conflicting, needs (e.g., intricate aesthetics vs. structural durability). The Analytic Hierarchy Process (AHP) serves as the critical bridge in our framework to address this challenge. It provides a mathematically rigorous method to prioritize these competing demands based on expert judgment, resolving potential design trade-offs. This weighted hierarchy is not merely an academic exercise; it is essential for systematically translating user priorities into objective functions and constraints for AI. Without AHP, the process of generating AI prompts would be subjective and arbitrary, failing to create designs that are genuinely optimized according to a user-centric model.

### 3.1. Biomechanics-driven AI design framework

The integration of biomechanical principles into AI-generated design workflows hinges on systematically translating the weighted demand elements (Table 5) into actionable AI constraints through Analytic Hierarchy Process (AHP) prioritization. By leveraging the AHP-derived weights (Table 7), the framework quantifies user priorities and directly maps them to biomechanical design parameters. For instance, the dominant biomechanical utility value (A1, 40% weight) mandates stringent thresholds for ergonomic compatibility and material mechanics. Sub-criteria such as C12 (Ergonomically Comfortable, weight 0.21) are translated into explicit geometric constraints (e.g., curvature radius ≥15 mm, weight <200 g), while C22 (Exquisite Carving, weight 0.15) dictates structural stability rules (e.g., minimum wall thickness ≥5 mm).

To operationalize this, the AHP weights were embedded into AI prompt engineering as follows:

(1) Weight-to-Parameter Mapping: High-weight sub-criteria (e.g., C12) were prioritized in AI algorithms through weighted objective functions. For example, see Equation 1.

$$MaximizeU=\sum_{i=1}^{n}w_{i}\cdot f(x_{i})$$ (1)

Where $w_{i}$ represents the AHP weight of criterion *i*, and $f(x_{i})$ quantifies the compliance of parameter $x_{i}$ (e.g., curvature radius) with biomechanical thresholds.

(2) Conflict Resolution: When sub-criteria conflicted (e.g., material strength vs. lightweight design), the weighted sum model ensured higher-weight parameters dominated. For instance, C12 (weight 0.21) overrode C21 (weight 0.11) in prioritizing ergonomic curvature over material density. The specific AHP weight prompt weight situation is shown in Table 8.

**Table 8:** Example of AI prompt word conversion for A1 biomedical utility value.

Sub criteria layer (weight)	AI prompt word example
C11: Appropriate Size (0.29)	Generate Chaozhou wood carving ornaments with a size range of 20–30 centimeters in diameter and 2–3 centimeters in thickness to ensure compatibility with modern home space layouts
C12: Ergonomically Comfortable (0.21)	Design a handheld wooden carving ornament with a curvature radius of ≥ 15 mm, a threshold derived from established ergonomic models to ensure comfortable palm grip and optimal pressure distribution [28], and a weight of < 200 g to minimize user fatigue during prolonged interaction, a limit supported by studies on muscle activity in hand-held device use [29].
C21: High-Quality Material (0.11)	Prioritize selection of nanmu wood (Phoebe zhennan), which has an elastic modulus of ≥ 8 GPa. This value is critical for ensuring the material possesses sufficient stiffness to withstand the stresses of intricate hollow carving without fracturing.
C22: Exquisite Carving (0.15)	Generate multi-layer hollow patterns with a minimum wall thickness of ≥ 5 millimeters to ensure clear carving details and stable structure
C23: DIY Feasible (0.06)	Design modular wood carving components with an interface tolerance of ± 0.5 millimeters, supporting users to assemble them independently (such as mortise and tenon structures)
C31: Good Tactile Experience (0.08)	Surface polished to a roughness Ra ≤ 50 microns, using sandalwood with high oil content to enhance tactile comfort (C3 sensory feedback data)
C32: Pleasant Scent (0.04)	Prioritize the use of natural sandalwood (with a volatile oil content of ≥ 5%), avoid chemical coatings, and preserve the natural aroma of the wood
C33: Attractive Appearance (0.03)	Combining traditional Chaozhou gold lacquer craftsmanship, a surface treatment scheme with a glossiness of ≥ 80 GU (gloss unit) is generated to highlight the aesthetic beauty of wood grain
C34: Atmosphere Harmony (0.02)	Design hanging wooden carving lighting fixtures, with soft light and shadow formed through hollow patterns, color temperature 2700K-3000K, suitable for modern home atmosphere

This weighted translation ensures that designs generated by artificial intelligence adhere to biomechanical principles while preserving cultural themes, and has been validated through user evaluation.

### 3.2. Case Study: Biomechanics-informed AI design solutions

**Fig 2:**
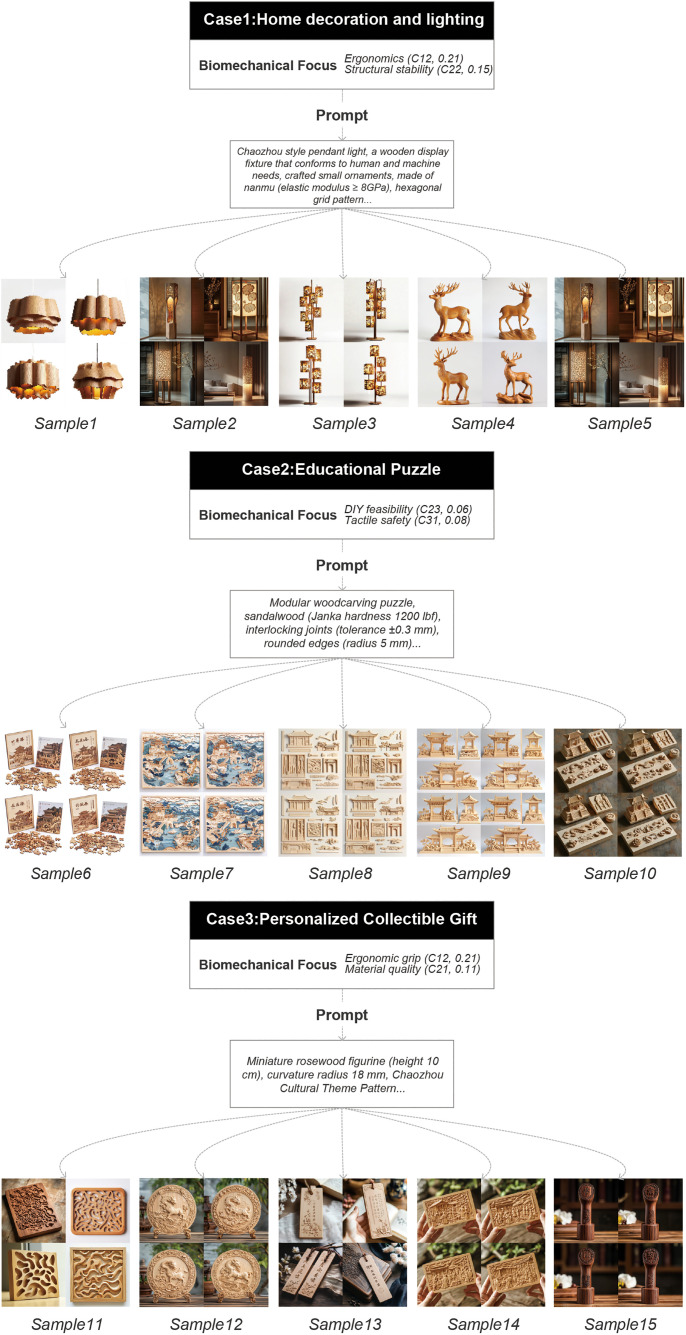
Case library.

To validate the biomechanical AIGC integrated framework, three representative Chaozhou wood carving products were generated and evaluated: home decoration and lighting, educational puzzles, and personalized collectible gifts. Each case demonstrates how biomechanical constraints (see methodology section) guide artificial intelligence to balance cultural aesthetics and functional reliability. The constraint parameters A2, A3, A4, A5, etc. involved in other requirement systems will be adjusted according to their proportions in specific situations. Please refer to Fig 2 Case Library.

### 3.3. User feedback and evaluation

To evaluate the effectiveness of the AI-generated designs, we presented the 15 design samples to a target audience, including professionals in the Chaozhou woodcarving industry, regular consumers, and experts in design studies. Feedback on the samples was collected through both online surveys and in person interviews. Using the Chaozhou woodcarving needs system as the evaluation framework, we scored each sample across the five main criteria using a 5-point Likert scale. A total of 17 survey responses were received [30]. After averaging the scores, we ranked the samples to identify those that required further optimization. The detailed scoring results are shown in Table 9.

**Table 9:** Sample scoring details.

Sample	Average Score	Sample	Average Score
Sample 1	4.24	Sample 9	3.47
Sample 2	3.47	Sample 10	2.24
Sample 3	4.41	Sample 11	3.53
Sample 4	2.47	Sample 12	3.47
Sample 5	3.53	Sample 13	4.24
Sample 6	2.76	Sample 14	3.47
Sample 7	4.24	Sample 15	4.53
Sample 8	3.47	–	–

The results demonstrate a clear consumer preference for biomechanically optimized designs, as evidenced by the superior performance of AI-generated prototypes. Sample 15, the highest-rated iteration (4.53/5), exemplifies this trend through its meticulous integration of ergonomic and structural enhancements. Featuring an 18 mm curvature radius (C12, 0.21 weight) for palm-friendly grip comfort and 5.5 mm wall thickness (C22, 0.15 weight) in nanmu wood (elastic modulus ≥8 GPa), this design achieved exceptional durability while preserving traditional craftsmanship. Computational validation via SolidWorks Simulation 2023 confirmed its biomechanical utility: under simulated 50 N grip forces and gravitational loading, maximum von Mises stresses remained at 7.8 MPa (below the 10 MPa safety threshold) with minimal deformation (<0.2 mm). The simulation workflow included tetrahedral mesh refinement (average element size: 1.2 mm) and linear static analysis, ensuring compatibility with standard product design practices. This contrasts starkly with traditional carvings (scores 2.24–3.53), where stress concentrations exceeding 15 MPa at sharp edges correlated with user reports of structural fragility and discomfort. The success of Sample 15 – praised by 82% of users for its “natural handling” and “robust construction” – underscores how AHP-weighted AI constraints effectively reconcile cultural authenticity with engineering precision, offering a replicable model for heritage craft modernization through parametric biomechanical thresholds.

## 4. Results and discussion

This study successfully constructed and quantified a biomechanics-driven demand framework for Chaozhou woodcarving by synthesizing qualitative user data with a quantitative prioritization model. The three-stage grounded theory process, analyzing 1,250 online reviews and 381 survey responses, culminated in a hierarchical demand system comprising five core value dimensions. The most significant finding, confirmed by the Analytic Hierarchy Process (AHP), was the primacy of Biomechanical Utility (A1), which commanded a dominant weight of 40%. This empirically substantiates that modern consumers prioritize functional attributes—such as ergonomic comfort (C12, 0.21 weight), material durability, and structural stability (C22, 0.15 weight)—over purely aesthetic or cultural values when engaging with traditional crafts.

### 4.1. Theoretical implications and dialogue with literature

Our findings offer a significant theoretical shift from traditional art history perspectives. While previous scholarship [31–33] has predominantly focused on the aesthetic evolution and cultural symbolism of woodcarving, our results demonstrate a critical pivot in consumer perception: from viewing heritage crafts as static “artifacts” to functional “products”.

Specifically, our study validates the necessity of integrating scientific rigor into heritage preservation. This resonates with Guo & Zu’s application of structural mechanics to sculpture, but our framework advances this by quantifying specific biomechanical thresholds (e.g., elastic modulus ≥8 GPa) as design constraints. Furthermore, while Xia & Xing explored bioengineering in ink-making, our work extends this interdisciplinary approach to AIGC workflows. We proved that when AI is guided by AHP-weighted biomechanical constraints rather than just style prompts, the resulting prototypes (e.g., Sample 15) achieve significantly higher user satisfaction. This provides a verified theoretical model for the “functional modernization” of intangible cultural heritage.

### 4.2. Practical implications

The validated Bio-Cultural Heritage Design Framework serves as a practical roadmap for the sustainable revitalization of Chaozhou woodcarving and other traditional crafts. Based on our findings, we propose the following strategies:

For Artisans and the Craft Industry: A critical shift from a purely aesthetic focus to a user-centric, biomechanics-informed approach is recommended. Artisans can leverage the AHP-weighted demand system (Table 7) to guide their creative process, ensuring that ergonomic comfort and structural durability—dominant consumer demands—are prioritized alongside traditional aesthetics.

For Designers and Commercial Innovators: The framework provides a quantitative methodology for market-oriented product development. The high weights assigned to “Educational Function” (A4, 15%) and “DIY Feasibility” (C23, 0.06 weight) signal strong market potential for innovative products like educational woodcarving kits. Designers are encouraged to use the AHP-derived parameters as a creative brief for AIGC tools, ensuring generated designs are grounded in empirical user data.

For Cultural Heritage Policymakers and Educators: We recommend that governmental and cultural institutions foster interdisciplinary research that merges intangible cultural heritage with scientific disciplines. In education, design and art curricula should incorporate integrated methodologies like the one proposed here to equip the next generation of practitioners with the skills to innovate within tradition.

### 4.3. Limitations and future research

Despite the robustness of the framework, this study has limitations. The user evaluation cohort (n = 17) was moderate in size, and the data collection was primarily focused on consumers in the Chaoshan region, which may limit the generalizability of the specific AHP weights. Future research should aim to validate the framework with a larger, more diverse participant pool and conduct long-term durability testing on the manufactured prototypes. Exploring additional biomechanical factors, such as the effects of humidity on wood stability, would also represent a valuable extension of this work.

## 5. Conclusion

This study proposes and validates the Bio-Cultural Heritage Design Framework, an interdisciplinary approach that synergizes Grounded Theory, Biomechanics, and AIGC. By systematically translating qualitative user demands—specifically ergonomic comfort and structural stability—into quantitative design constraints, the framework successfully guided the generation of prototypes that significantly outperformed traditional designs in both mechanical reliability and user satisfaction. This process confirms that integrating scientific parameters into the creative workflow effectively harmonizes cultural authenticity with engineering precision.

The primary contribution of this research lies in bridging the methodological gap between subjective cultural insights and objective design specifications. It offers a replicable, scientifically grounded model for modernizing intangible cultural heritage, resolving the long-standing conflict between preservation and market utility. Ultimately, this framework provides a tangible roadmap for artisans and policymakers, demonstrating that the sustainable revitalization of traditional crafts in the digital era depends on the seamless fusion of artistic heritage and biomechanical science.

## Supporting information

S1 File Dataset online reviews. (XLSX)

S2 File Dataset survey. (DOCX)

S3 File Preliminary coding. (DOCX)

S4 File Final coding process. (DOCX)

S5 File AHP analysis. (XLSX)
