# Supplementary material for: Research on the development of Chaozhou woodcarving from the perspectives of grounded theory and biomechanics
Source: PLoS One. 2026 Feb 18;21(2):e0341450. doi: 10.1371/journal.pone.0341450 (PMC12915953; doi:10.1371/journal.pone.0341450)
Supplement: S2 File — (DOCX) [file pone.0341450.s002.docx]

**Chaozhou Woodcarving Home Product Design Demand Survey Questionnaire**

1. Your gender is: [Single choice question]

| option | subtotal | proportion |
| --- | --- | --- |
| male | 182 | 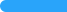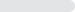47.77% |
| female | 199 | 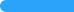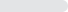52.23% |
| Number of valid respondents for this question | 381 |  |

2. Your age is: [Single choice question]

| option | subtotal | proportion |
| --- | --- | --- |
| under 18 | 5 | 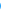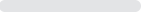1.31% |
| 18-24 years old | 128 | 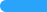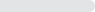33.6% |
| 25-34 years old | 120 | 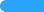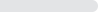31.5% |
| 35-44 years old | 81 | 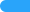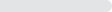21.26% |
| 45 years old and above | 47 | 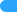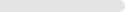12.34% |
| Number of valid respondents for this question | 381 |  |

1. Your occupation is: [Single choice question]

| option | subtotal | proportion |
| --- | --- | --- |
| student | 17 | 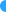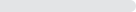4.46% |
| White collar/office staff | 253 | 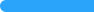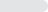66.4% |
| freelancer | 38 | 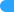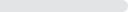9.97% |
| Housewife/Husband | 55 | 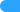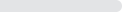14.44% |
| other: | 18 | 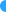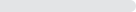4.72% |
| Number of valid respondents for this question | 381 |  |

4. Your monthly income range is: [Single choice question]

| option | subtotal | proportion |
| --- | --- | --- |
| no income | 36 | 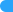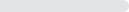9.45% |
| Below 3000 yuan | 53 | 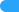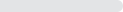13.91% |
| 3000-5000 yuan | 81 | 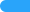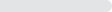21.26% |
| 5000-10000 yuan | 122 | 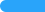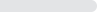32.02% |
| Over 10000 yuan | 89 | 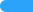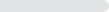23.36% |
| Number of valid respondents for this question | 381 |  |

5. Your understanding of Chaozhou wood carving culture is: [Single choice question]

| option | subtotal | proportion |
| --- | --- | --- |
| very familiar with | 343 | 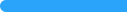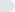90.03% |
| A little understanding | 38 | 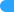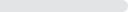9.97% |
| Number of valid respondents for this question | 381 |  |

6. The main purposes for which you currently purchase household products are: [Single choice question]

| option | subtotal | proportion |
| --- | --- | --- |
| Daily practicality (wooden lamps, photo frames, wooden carvings) | 163 | 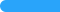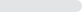42.78% |
| Decorative embellishments (wood carvings, wooden crafts, flower and bird sculptures) | 111 | 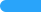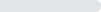29.13% |
| Gift DIY (mini carved artworks, makeup jewelry, care products) | 55 | 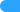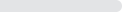14.44% |
| Faith (Buddhist niche, wooden Buddha statue, jewelry pendant) | 42 | 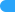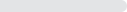11.02% |
| Other:_________ | 10 | 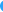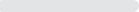2.62% |
| Number of valid respondents for this question | 381 |  |

7. Which of the following features do you value more in home products? (Multiple Choice) [Multiple Choice Question]

| option | subtotal | proportion |
| --- | --- | --- |
| aesthetics | 184 | 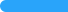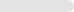48.29% |
| practicality | 261 | 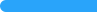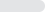68.5% |
| comfort | 193 | 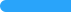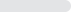50.66% |
| cultural value | 173 | 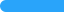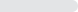45.41% |
| Uniqueness/Personalization | 226 | 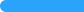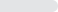59.32% |
| Number of valid respondents for this question | 381 |  |

8. What application methods of Chaozhou wood carving do you hope to see in home products? (Multiple Choice) [Multiple Choice Question]

| option | subtotal | proportion |
| --- | --- | --- |
| Furniture (such as tables, chairs, cabinets) | 325 | 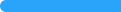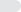85.3% |
| Decorations (such as pendants, ornaments) | 279 | 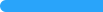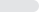73.23% |
| Daily necessities (such as lamps, mirrors) | 175 | 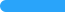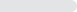45.93% |
| other | 4 | 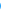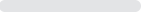1.05% |
| Number of valid respondents for this question | 381 |  |

9.Are you willing to pay higher prices for home products to obtain cultural heritage or customized services? Single Choice Question

| option | subtotal | proportion |
| --- | --- | --- |
| yes | 369 | 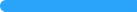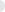96.85% |
| No | 12 | 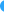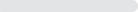3.15% |
| Number of valid respondents for this question | 381 |  |

10. If Chaozhou woodcarving is combined with modern design, which style would you prefer? Single Choice Question

| option | subtotal | proportion |
| --- | --- | --- |
| Traditional Chinese style | 111 | 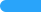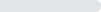29.13% |
| New Chinese Style | 139 | 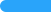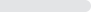36.48% |
| modern style | 92 | 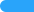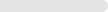24.15% |
| Chinese and Nordic mixed style | 33 | 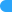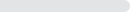8.66% |
| other | 6 | 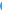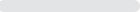1.57% |
| Number of valid respondents for this question | 381 |  |

11.What are the characteristics of Chaozhou wood carving that you prefer? (Multiple Choice) [Multiple Choice Question]

| option | subtotal | proportion |
| --- | --- | --- |
| Delicate carving | 303 | 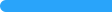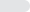79.53% |
| Patterns rich in cultural symbols | 254 | 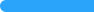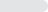66.67% |
| The uniqueness of handicrafts | 232 | 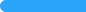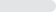60.89% |
| The high-end feel of the material | 182 | 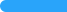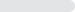47.77% |
| Smooth lines | 138 | 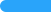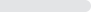36.22% |
| Vivid and vivid imagery | 320 | 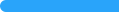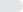83.99% |
| other | 6 | 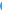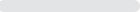1.57% |
| Number of valid respondents for this question | 381 |  |

12. If you want to buy Chaozhou household products, your household budget is generally: [Single choice question]

| option | subtotal | proportion |
| --- | --- | --- |
| under 500 yuan | 72 | 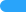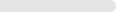18.9% |
| 500-1000 yuan | 148 | 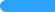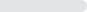38.85% |
| 1000-3000 yuan | 134 | 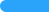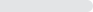35.17% |
| Over 3000 yuan | 27 | 7.09% |
| Number of valid respondents for this question | 381 |  |

13. The main channels for you to purchase household products are: [Single choice question]

| option | subtotal | proportion |
| --- | --- | --- |
| Online e-commerce platforms (such as Taobao, JD.com) | 127 | 33.33% |
| -Offline home shopping mall | 162 | 42.52% |
| Handicraft exhibitions or specialty stores | 91 | 23.88% |
| other: | 1 | 0.26% |
| Number of valid respondents for this question | 381 |  |

14.Would you consider customizing a home product with Chaozhou wood carving elements? Single Choice Question

| option | subtotal | proportion |
| --- | --- | --- |
| can | 120 | 31.5% |
| won't | 36 | 9.45% |
| Depending on the price | 225 | 59.06% |
| Number of valid respondents for this question | 381 |  |

15.If you could customize a home product with Chaozhou wood carving elements, what would you most like it to be? Please briefly describe: [fill in the blank question]

carve

Rich culture

Small and delicate flowers

Carving Zodiac Images Gathering

Show solemnity

Carving lamp

Wooden carving frame

chair

Texture anti slip

sofa

pattern

Independence of the Atmosphere Country

Wooden carving vase

elegant

Carving simple lines

Chaozhou wood carving

The wind pattern is simple

Wooden carved chessboard

Carved with plant patterns

Practical patterns

Rich cultural heritage

Add warmth

Place fruits

Beautiful decoration

Dining is more stylish

More artistic and unique

Carved with phoenix pattern

smooth lines
