# Supplementary material for: Research on the development of Chaozhou woodcarving from the perspectives of grounded theory and biomechanics
Source: PLoS One. 2026 Feb 18;21(2):e0341450. doi: 10.1371/journal.pone.0341450 (PMC12915953; doi:10.1371/journal.pone.0341450)
Supplement: S3 File — (DOCX) [file pone.0341450.s003.docx]

| 2026/1/10 17:34 | | | | |
| --- | --- | --- | --- | --- |
| Node Structure | | | | |
| Chaozhou Woodcarving | | | | |
| 2026/1/10 17:34 | | | | |
| **Hierarchical Name** | | **Nickname** | **Total** | **User-assigned Color** |
| **Node** | | | | |
| **Node\JD.com Code** | | | | |
| Node\JD.com Code\Ornament | |  | Yes | None |
| Node\JD.com Code\Ornament\Feng Shui Ornament | |  | No | None |
| Node\JD.com Code\Ornament\Scratched Woodcarving Ornament | |  | No | None |
| Node\JD.com Code\Ornament\Prize Woodcarving Ornament | |  | No | None |
| Node\JD.com Code\Ornament\Gift Box Woodcarving Ornament | |  | No | None |
| Node\JD.com Code\Ornament\Cat Woodcarving | |  | No | None |
| Node\JD.com Code\Ornament\Small Ornament | |  | No | None |
| Node\\JD.com Coding\\Ornament\Small Woodcarving | |  | No | None |
| Node\\JD.com Coding\\Ornament\Desktop Ornament | |  | No | None |
| Node\\JD.com Coding\\Packaging | |  | Yes | None |
| Node\\JD.com Coding\\Packaging\Packaging Bags | |  | No | None |
| Node\\JD.com Coding\\Packaging\Authentic Packaging | |  | No | None |
| Node\\JD.com Coding\\Packaging\Elegant Packaging | |  | No | None |
| Node\\JD.com Coding\\Packaging\Hairpin Packaging Box | |  | No | None |
| Node\\JD.com Coding\\Packaging\Intact Internal and External Packaging | |  | No | None |
| Node\\JD.com Coding\\Packaging\Standard Packaging | |  | No | None |
| Node\\JD.com Coding\\Carving | |  | Yes | None |
| Node\\JD.com Coding\\Carving\Carving Quality | |  | No | None |
| Node\\JD.com Coding\\Carving\Superior Carving Craftsmanship | |  | No | None |
| Node\JD.com Coding\Carving\Carving Patterns | |  | No | None |
| Node\JD.com Coding\Carving\Delicate Carving | |  | No | None |
| Node\JD.com Coding\Carving\Detailed Carving | |  | No | None |
| Node\JD.com Coding\Carving\Fine Carving | |  | No | None |
| Node\JD.com Coding\Carving\Machine Carving | |  | No | None |
| Node\JD.com Coding\Carving\Sword Blade Carving | |  | No | None |
| Node\JD.com Coding\Carving\Wrist Mala Carving | |  | No | None |
| Node\JD.com Coding\Carving\Texture Carving | |  | No | None |
| Node\JD.com Coding\Shopping | |  | Yes | None |
| Node\JD.com Coding\Shopping\Shopping Process | |  | No | None |
| Node\JD.com Coding\Shopping\Shopping Experience | |  | No | None |
| Node\JD.com's Coding\Shopping\User Experience | |  | No | None |
| Node\JD.com's Coding\Good | |  | Yes | None |
|  | | | | |
| Report\Node Structure Report | Page 1 of 3 | | | |
| 2026/1/10 17:34 | | | | |
| **Hierarchical Name** | | **Nickname** | **Total** | **User-assigned Color** |
| Node\JD.com's Coding\Good\Good Dragon | |  | No | None |
| Node\JD.com's Coding\Good\Good Twelve | |  | No | None |
| Node\JD.com's Coding\Good\Good Carving | |  | No | None |
| Node\JD.com's Coding\Good\Good Items | |  | No | None |
| Node\JD.com's Coding\Good\Good Friends | |  | No | None |
| Node\JD.com's Coding\Good\Positive Reviews | |  | No | None |
| Node\JD.com's Coding\Good\Recommendations | |  | No | None |
| Node\JD.com's Coding\Good\Physical Stores' Quality | |  | No | None |
| Node\JD.com's Coding\Gifts | |  | Yes | None |
| Node\JD.com's Coding\Gifts\Others' Gifts | |  | No | None |
| Node\JD.com's Coding\Gifts\Husband's Gifts | |  | No | None |
| Node\\JD.com Coding\\Gifts\Gift for a Person | |  | No | None |
| Node\\JD.com Coding\\Gifts\Authentic Gift | |  | No | None |
| Node\\JD.com Coding\\Gifts\Girlfriend's Gift | |  | No | None |
| Node\\JD.com Coding\\Gifts\Christmas Gift | |  | No | None |
| Node\\JD.com Coding\\Gifts\Gift for Presentation | |  | No | None |
| Node\\JD.com Coding\\Gifts\Small Gift | |  | No | None |
| Node\\JD.com Coding\\Cat | |  | Yes | None |
| Node\\JD.com Coding\\Cat\Dotted Walking Cat | |  | No | None |
| Node\\JD.com Coding\\Cat\Cat Face | |  | No | None |
| Node\\JD.com Coding\\Cat\Cat Woodcarving | |  | No | None |
| Node\\JD.com Coding\\Cat\Cat Posture | |  | No | None |
| Node\JD.com's Coding\Cat\Lacquered Strolling Cat | |  | No | None |
| Node\JD.com's Coding\Cat\Kitten | |  | No | None |
| Node\JD.com's Coding\Small | |  | Yes | None |
| Node\JD.com's Coding\Small\Size | |  | No | None |
| Node\JD.com's Coding\Small\Small Squint | |  | No | None |
| Node\JD.com's Coding\Small\Small Ornament | |  | No | None |
| Node\JD.com's Coding\Small\Small Carving | |  | No | None |
| Node\JD.com's Coding\Small\Small Item | |  | No | None |
| Node\JD.com's Coding\Small\Small Animal | |  | No | None |
| Node\JD.com's Coding\Small\Small Gift | |  | No | None |
| Node\JD.com's Coding\Small\Kitten | |  | No | None |
| Node\JD.com's Coding\Small\Kitten Carving | |  | No | None |
| Node\JD.com's Coding\Small\Little Kitten | |  | No | None |
| Node\JD.com's Coding\Small\Cute Little Animal | |  | No | None |
| Node\JD.com Coding\Small\Kitten | |  | No | None |
| Node\JD.com Coding\Small\Small Woodcarving | |  | No | None |
| Node\JD.com Coding\Small\Little Friend | |  | No | None |
| Node\JD.com Coding\Small\Small Ornament | |  | No | None |
| Node\JD.com Coding\Small\Small Gloves | |  | No | None |
| Node\JD.com Coding\Small\Small Sandalwood | |  | No | None |
|  | | | | |
| Report\Node Structure Report | Page 2 of 3 | | | |
| 2026/1/10 17:34 | | | | |
| **Hierarchical Name** | | **Nickname** | **Total** | **User-assigned Color** |
| Node\JD.com Coding\Small\Small Trinket | |  | No | None |
| Node\JD.com Coding\Small\Small Pinch | |  | No | None |
| Node\JD.com Coding\Kitten | |  | Yes | None |
| Node\JD.com Coding\Kitten\Kitten | |  | No | None |
|  | | | | |
| **Node\Manual Coding** | | | | |
| Node\Manual Coding\Fidgeting | |  | No | None |
| Node\Manual Coding\Ornament | |  | No | None |
| Node\Manual Coding\Material and Craftsmanship | |  | No | None |
| Node\Manual Coding\Texture | |  | No | None |
| Node\Manual Coding\Size | |  | No | None |
| Node\Manual Coding\Grade | |  | No | None |
| Node\Manual Coding\Animal | |  | No | None |
| Node\Manual Coding\Atmosphere | |  | No | None |
| Node\Manual Coding\Feng Shui | |  | No | None |
| Node\Manual Coding\Feng Shui\Good Luck | |  | No | None |
| Node\Manual Coding\Feng Shui\Evil Spirit Protection | |  | No | None |
| Node\Manual Coding\Shopping Experience | |  | No | None |
| Node\Manual Coding\Official Recognition | |  | No | None |
| Node\Manual Coding\Price Range | |  | No | None |
| Node\Manual Coding\Education | |  | No | None |
| Node\Manual Coding\Stress Relief | |  | No | None |
| Node\Manual Coding\Cute | |  | No | None |
| Node\Manual Coding\Gift Packaging | |  | No | None |
| Node\Manual Coding\Satisfaction | |  | No | None |
| Node\Manual Coding\Scent | |  | No | None |
| Node\Manual Coding\Human-Machine Scale | |  | No | None |
| Node\Manual Coding\Handling Difficulty | |  | No | None |
| Node\Manual Coding\Design Sense | |  | No | None |
| Node\Manual Coding\Expression | |  | No | None |
| Node\Manual Coding\Deity | |  | No | None |
| Node\Manual Coding\Deity\Buddha | |  | No | None |
| Node\Manual Coding\Ornament | |  | No | None |
| Node\Manual Coding\Visual | |  | No | None |
| Node\Manual Coding\Wrist Mala | |  | No | None |
| Node\Manual Coding\Interests and Hobbies | |  | No | None |
| Node\Manual Coding\Color | |  | No | None |
|  | | | | |
|  | | | | |
| Report\Node Structure Report | Page 3 of 3 | | | |
