# Supplementary material for: Research on the development of Chaozhou woodcarving from the perspectives of grounded theory and biomechanics
Source: PLoS One. 2026 Feb 18;21(2):e0341450. doi: 10.1371/journal.pone.0341450 (PMC12915953; doi:10.1371/journal.pone.0341450)
Supplement: S4 File — (DOCX) [file pone.0341450.s004.docx]

Table 2. Open Encoding

| **Initial Category** | **Initial Concept** | **Original Statement (Example)** |
| --- | --- | --- |
| C1  Form and Ergonomics Fit | a1 Ergonomic Woodcarving Ornament  a2 Ergonomic Woodcarving Handle Piece  a3 Ergonomic Woodcarving Jewelry  a4 Ergonomic Woodcarving Bead String  a5 Ergonomic Woodcarving Creative Item  a6 Ergonomic Compatibility | The unique texture and lightweight design of the solid wood make it an ideal ergonomic ornament, fitting comfortably in various settings.  The vivid carving and compact size create an adorable ergonomic handle piece, perfect for enhancing the comfort of a tea table setup.  The beautiful bead string features ergonomic jewelry design with a size that fits comfortably when worn, ensuring both aesthetics and comfort.  The lightweight design of the bird figurine ensures it has good ergonomic compatibility, making it easy to place and reposition.  The exquisite bird figurine, with its palm-sized dimensions, serves as an ergonomic decorative item that fits well in various spaces. |
| C2 Material Mechanics and Craftsmanship | a7 Carving Craftsmanship and Material Strength  a8 Wood Material Mechanical Properties  a9 DIY Creation and Material Process ability  a10 Craftsmanship Difficulty and Material Mastery | The beads exhibit uniform size and smoothness, showcasing excellent carving craftsmanship that enhances the material's strength and provides a superior tactile experience.  Carving the hairpin required three hours, demonstrating the material's process ability and the fine craftsmanship achieved, which I appreciate and will repurchase for its quality.  Mastering the material's properties through skillful manual carving is quite challenging, and completing it signifies a meaningful achievement in craftsmanship difficulty and material mastery. |
| C3 Sensory Feedback during Use | a11 Tactile Experience during Use  a12 Aroma Experience during Use  a13 Visual Appeal during Use  a14 Atmosphere Created by the Product | The aged wood has a faint scent of sandalwood and high oil content, giving a strong sense of weight when held. Each bead is plump, round, and smooth, providing a great tactile experience during use.  I'm extremely satisfied with the material's tactile experience and the color tone. The small-leaf rosewood has a wonderful aroma, and when I add a few drops of my essential oil, the sensory experience during use is just perfect. |
| C4 Aesthetic Style | a15 Design Aesthetics  a16 Artistic Style  a17 Creativity and Novelty | The seller was really great, and I picked a nice string of beads as a gift for someone. It's a Chinese zodiac bracelet, very traditional and Chinese in style. I'm very satisfied!  The beads are very beautiful, with unique shapes. The item has a particularly trendy and fashionable style, and it looks great. |
| C5 Cultural Symbolism | a18 Cultural Symbolic Elements  a19 Feng Shui Implications  a20 Story Connotations | Displaying a peach wood sword at home not only demonstrates the owner's respect for traditional culture but also serves as a feng shui ornament, adding a touch of classical charm to the living environment. It's really quite nice.  I've received the Wen Chang Pagoda. I bought it to help my child with their studies, hoping they will achieve good grades and get into a good university. |
| C6 Shopping Attributes | a21 Gift-Giving Purpose  a22 Product Class  a23 Elegant Packaging  a24 After-Sales Service | After being sealed for a few days and then taken out for handling, I simply love it. The beads are oily and smooth with plenty of gold stars, and the gifts included are also rich and practical.  The birthday gift for my friend has arrived, and I'm very satisfied. The craftsmanship is very meticulous and not at all rough. |
| C7 Value Assessment | a25 Value Assessment  a26 Price Reasonableness  a27 Cost-Effectiveness | The bead string is really good, and I'm very satisfied at this price point.  It has a high aesthetic value and looks really great when worn. The cost-effectiveness is truly exceptional. |
| C8 Educational Function | a28 Educational Inspiration  a29 Heritage Carrier  a30 Hands-on Practice | It was so much fun! I sat down with my kid for the whole afternoon with the tools. |
| C9 Psychological Needs | a31 Stress-Relief Effect  a32 Emotional Appeal  a33 Collecting Intention | Whittling wood is very relaxing. Friends who are interested should definitely give it a try.  I spotted this adorable little figurine at first glance and bought it as a decoration for my girlfriend. She really loves it! It’s such a cute little cat! I believe it will bring good luck for the New Year! |

Table 3. Axial Coding of Main Categories and Corresponding Initial Categories

| **Main Category** | **Initial Category** | **Original Statement (Example)** |
| --- | --- | --- |
| A1 Biomechanical Utility Value | C1 Form and Ergonomics Fit  C2 Material Mechanics and Craftsmanship  C3 Sensory Feedback during Use | The design is unique and the texture is great, making it particularly suitable for placement in the car. The color matches the interior, and the size is just right.  The craftsmanship is exquisite, and the gifts included are also great. I bought it for my daughter, and it was a very satisfying purchase. The product has a good sensory feedback during use, making it a delightful experience overall. |
| A2  Cultural Aesthetic Value | C4 Aesthetic Style  C5 Cultural Symbolism  s | The Chinese-style zodiac bracelet is very beautiful when worn, and the size fits perfectly.  The craftsmanship is exquisite, and I hope it will bring my child good luck in their studies.  The peach wood sword is well-made. I got it to protect my home. The fine craftsmanship gives me peace of mind. |
| A3 Consumption and Social Value | C6 Shopping Attributes  C7 Value Assessment | The craftsmanship is exquisite, and the gifts included are also great. I bought it for my daughter, and it was a very satisfying purchase.  I bought it as a gift for my dad. He said it's very worth it at this price and he particularly likes it. |
| A4 Educational and Heritage Value | C8 Educational Function | It's really great, worth the money, and relaxing. Playing with my child was amazing. We not only learned how to use our hands but also enjoyed many vivid stories together. |
| A5 Emotional Fulfillment Value | C9 Psychological Need | It's very nice and soothing. The simple expression of the little cat captures a sense of contentment, reminding us that happiness can be found in simplicity. |

**Table 4.** Selective Encoding

| Typical Relationship Structure | Relationship Structure | Original Statement (Example) |
| --- | --- | --- |
| Biomechanical Utility  Affects Emotional Evaluation | A1 Biomechanical Utility Value  (Perceived Functional Quality) → A5 Emotional Fulfillment Value (Perceived Emotional Quality) | The craftsmanship is exquisite and suitable as a decoration; it looks very classy in the living room. I initially thought this piece would be larger, but upon receiving it, I realized the size is not quite right. It feels awkward to hold, and I'm not very fond of it. |
| Emotional Drive Influences Social Choice | A5 Emotional Fulfillment Value (Perceived Emotional Quality) → A3 Consumption and Social Value (Perceived Social Quality) | This little bird pendant is so cute!! I hope it brings me good luck, and I'll buy a few more to give to my friends. |
| Biomechanical Utility Feedbacks Social Interaction | A1 Biomechanical Utility Value (Perceived Functional Quality) → A3 Consumption and Social Value (Perceived Social Quality) | I carved this for my girlfriend for the first time, and she couldn't stop praising it. We even discussed together how to display it better. Ha haha, I'll buy another one to play with her. |
| Cultural Aesthetics Enrich Emotional Connotations | A2 Cultural Aesthetic Value → A5 Emotional Fulfillment Value | The solemn and beautiful deity statue, worshipped with a sincere heart, not only looks beautiful at home but also helps me maintain a compassionate mind and stay away from negative thoughts! |
| Educational Heritage Enhances Cultural Aesthetics | A4 Educational and Heritage Value → A2 Cultural Aesthetic Value | I bought it to play with my child, and only then did I truly understand the stories behind the woodcarvings. Now, when I look at those woodcarvings again, I find their beauty even more profound. |
